# Gene and lncRNA co-expression network analysis reveals novel ceRNA network for triple-negative breast cancer

**DOI:** 10.1038/s41598-019-51626-7

**Published:** 2019-10-22

**Authors:** Kehao Le, Hui Guo, Qiulei Zhang, Xiaojuan Huang, Ming Xu, Ziwei Huang, Pengfei Yi

**Affiliations:** 1 Department of Breast and Thyroid Surgery, Union Hospital, Tongji Medical College, Huazhong University of Science and Technology, Wuhan, China; 2 Department of Nuclear Medicine, Union Hospital, Tongji Medical College, Huazhong University of Science and Technology, Wuhan, China

**Keywords:** Biomarkers, Cancer

## Abstract

Breast cancer is the most frequently diagnosed malignancy among women, and triple-negative breast cancer (TNBC) is a highly aggressive subtype. Increasing evidence has shown that lncRNAs are involved in tumor growth, cell-cycle, and apoptosis through interactions with miRNAs or mRNAs. However, there is still limited data on ceRNAs involved in the molecular mechanisms underlying TNBC. In this study, we applied the weighted gene co-expression network analysis to the existing microarray mRNA and lncRNA expression data obtained from the breast tissues of TNBC patients to find the hub genes and lncRNAs involved in TNBC. Functional enrichment was performed on the module that correlated with Ki-67 status the most (Turquoise module). The hub genes in the Turquoise module were found to be associated with DNA repair, cell proliferation, and the p53 signaling pathway. We performed co-expression analysis of the protein-coding and lncRNA hub genes in the Turquoise module. Analysis of the RNA-seq data obtained from The Cancer Genome Atlas database revealed that the protein-coding genes and lncRNAs that were co-expressed were also differentially expressed in the TNBC tissues compared with the normal mammary tissues. On the basis of establishing the ceRNA network, two mRNAs (RAD51AP1 and TYMS) were found to be correlated with overall survival in TNBC. These results suggest that TNBC-specific mRNA and lncRNAs may participate in a complex ceRNA network, which represents a potential therapeutic target for the treatment of TNBC.

## Introduction

Breast cancer is the fifth leading cause of death and most frequently diagnosed malignancy in women worldwide^1^. It is characterized by at least four different clinically relevant molecular subtypes: Luminal A, Luminal B, her2-enriched type, and triple negative breast cancer (TNBC)^2^. TNBC is generally classified into basal-like and Claudin-low categories^3^. Due to the lack of expression of estrogen and progesterone receptors and HER2 amplification, there is no targeted therapy for this highly invasive breast cancer subtype^4^. In addition, TNBC presents with higher aggressiveness and poorer prognosis than the other subtypes as evidenced by the lower survival rate and increased risk of metastasis and recurrence in TNBC^5,6^. Therefore, the molecular mechanisms underlying TNBC should be further studied.

Long noncoding RNAs (lncRNAs) are defined as RNA transcripts ≥200 nucleotides long that do not encode for a protein^7^. In recent years, increasing evidence has shown that lncRNAs are involved in tumor growth, cell-cycle, and apoptosis through interactions with miRNAs or mRNAs^8–10^. In 2011, Salmena *et al*. proposed the concept of competing endogenous RNAs (ceRNAs), a class of RNAs with miRNA binding sites with which miRNA-targeted RNAs compete for miRNAs, and thus elucidated a complex post-transcriptional regulatory network including mRNAs, lncRNAs, and other types of RNAs^11^. LncRNAs can regulate gene expression by interacting with miRNAs. Several studies have confirmed this concept^12,13^. However, there has been few studies on the involvement of ceRNA-mediated mechanisms in TNBC.

Weighted gene co-expression network analysis (WGCNA) is a systematic in silico method for the analysis of complex gene regulatory networks and is based on gene expression data. WGCNA can be used to study biological networks based on genetic correlations. It identifies modules (clusters) of highly correlated genes^14^. By constructing correlation networks, WGCNA can identify candidate biomarkers and therapeutic targets for different types of cancer^15–18^. For instance, a few of hub genes associated with the pathological stage of colon cancer have been obtained by using this method^19^. In addition, Liu *et al*.^20^ have identified gene modules that constitute a recurrence-associated network.

In this study, we applied WGCNA in combination with functional enrichment analysis to the available TNBC mRNA and lncRNA expression data to identify the hub genes, including lncRNAs involved in TNBC. Verification and further analysis of the identified protein-coding genes and lncRNAs were conducted using The Cancer Genome Atlas (TCGA, https://tcga-data.nci.nih.gov/tcga/) database. As a result, 4,565 mRNAs, 427 miRNAs, and 4,852 lncRNAs were identified. Finally, 37 lncRNAs, 28 miRNAs, and 16 mRNAs were selected to construct a lncRNA-miRNA-mRNA ceRNA network. The major aim of this study is to discuss the molecular mechanisms underlying TNBC and provide novel prognostic factors for TNBC.

## Results

### Differential mRNA and lncRNA expression

**Figure 1:**
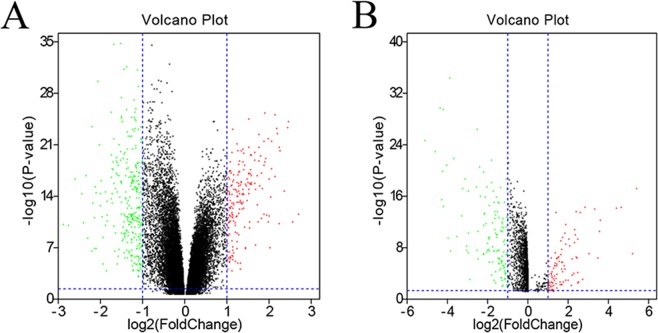
Differentially expressed protein-coding genes and lncRNAs. The volcano plot of the differentially expressed protein-coding genes (**A**) and lncRNAs (**B**) in normal and TNBC tissue samples in the GSE76250 dataset. Log_2_ fold change (cut-off = ±1, vertical lines) was plotted against the −log_10_ *p*-value (cut-off = 1.3, horizontal line).

The GSE76250 database includes 17,643 mRNAs from 198 samples, and GSE65216 database includes 2097 lncRNAs from 66 samples. In total, 9346 differentially expressed genes (DEGs) and 2097 lncRNAs (with *P*-value < 0.05) were screened out. The volcano plots of these mRNAs and lncRNAs are shown in Fig. 1.

### Identification of the gene co-expression modules

**Figure 2:**
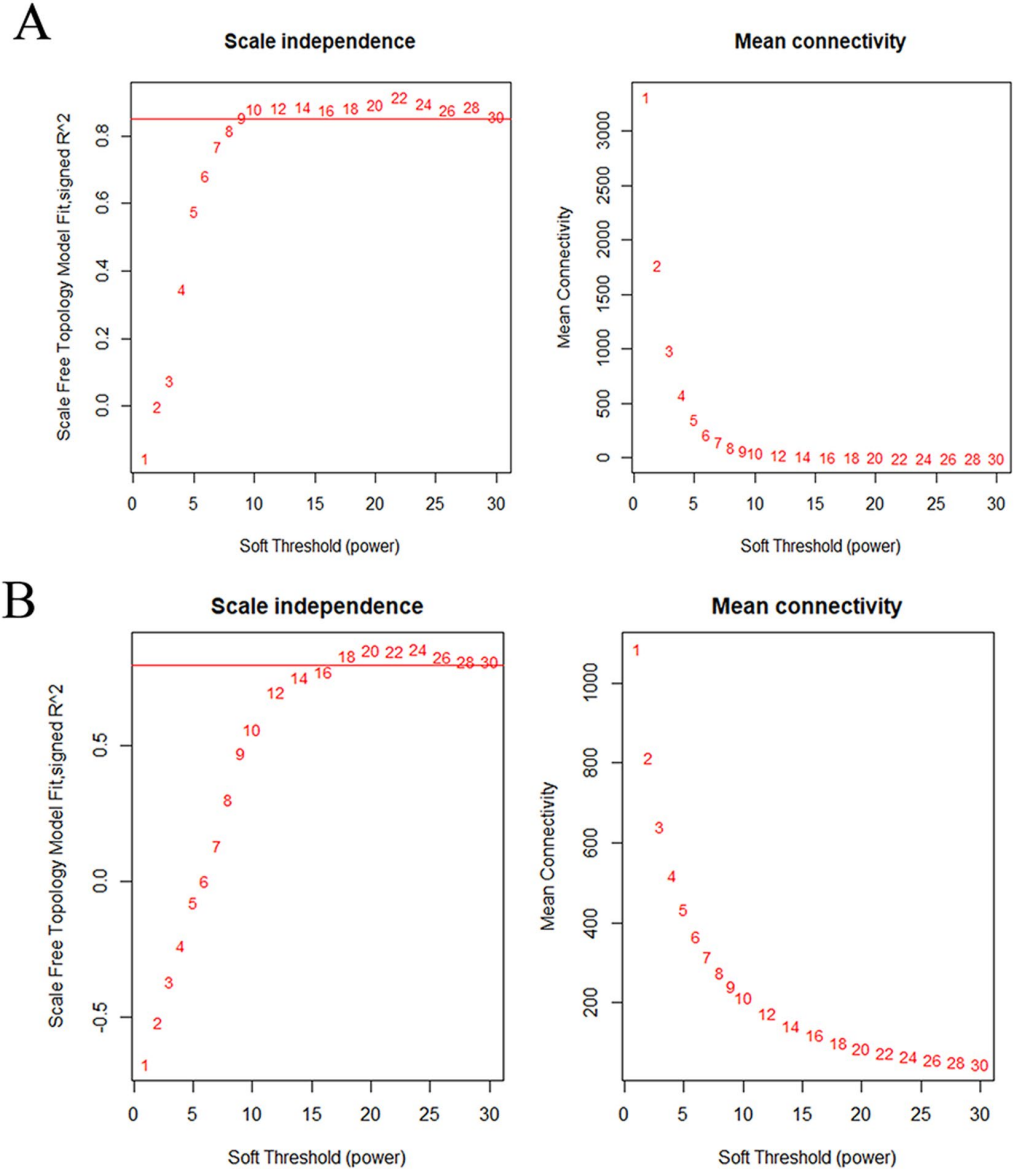
Analysis of the network topology for various soft-thresholding powers for the protein-coding gene subset (**A**) and the lncRNA subset (**B**). The left panels show the Scale-free Topology Fit Index (R2, y-axis) as a function of the soft-thresholding power (x-axis). The red line indicates an R2 of 0.85. The right panels display the mean connectivity (degree, y-axis) as a function of the soft-thresholding power (x-axis). All the networks have a correct scale-free topology since the Scale-free Topology Fit Index reached >0.85 for low powers (<30) for all the expression subsets: 9 for (**A**), 16–18 for (**B**).

**Figure 3:**
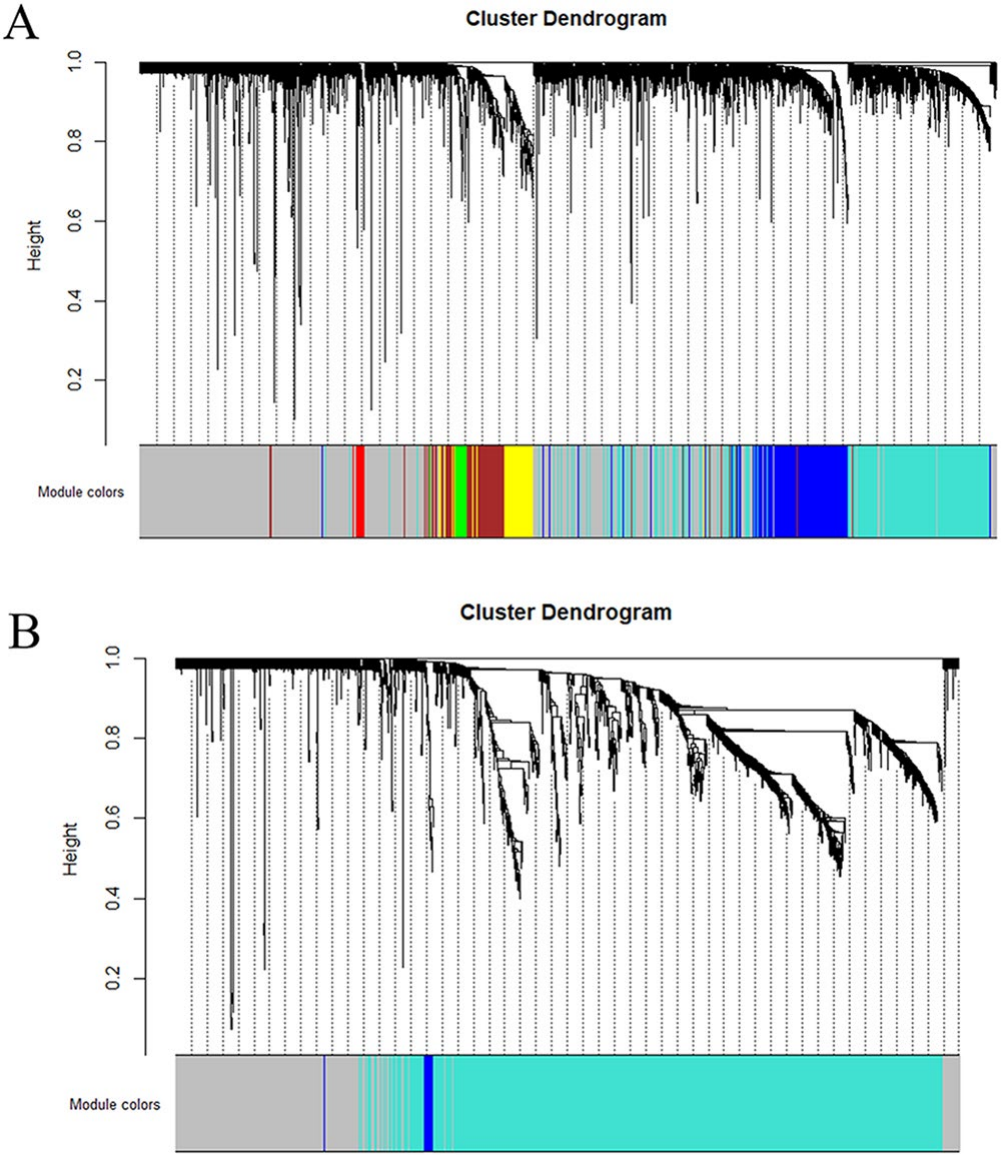
The clustering dendrograms and modules identified by WGCNA. (**A**) The clustering diagram and 9 modules for the protein-coding gene dataset. (**B**) The clustering diagram and 3 modules for the lncRNA dataset imposed on the network.

**Figure 4:**
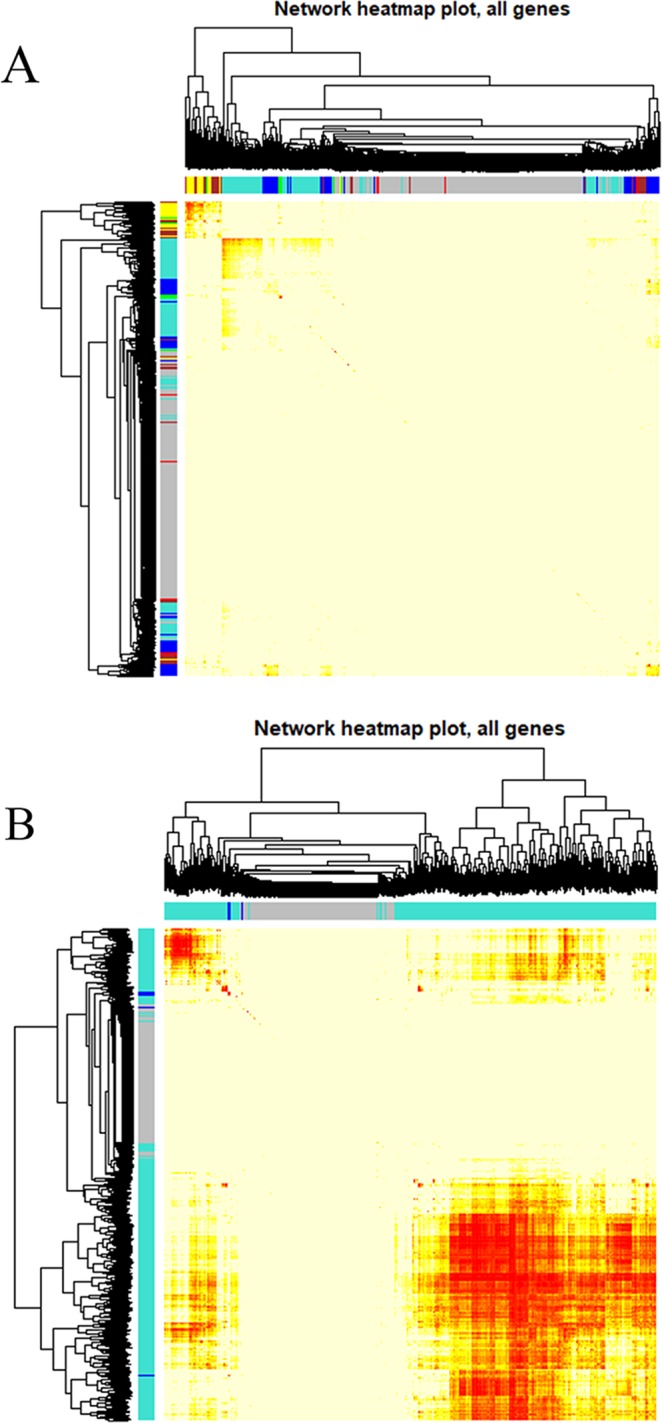
Heatmap plot representing the protein-coding gene network (**A**) and lncRNA network. (**B**) The heatmap depicts the topological overlap matrix among all the protein-coding genes and lncRNAs in the analysis.

To construct co-expression gene networks, we used the 9346 DEGs and 165 samples. The power value is the most critical parameter in WGCNA and mainly affects the mean connectivity and independence of the co-expression modules. Figure 2A shows that when the power reached 9, the scale-free topology fit index was 0.85. We then calculated the topological overlap matrix (TOM), which represents the connectivity of each gene in the network, for each mRNA pair. In all, by using the dynamic tree cut method, 9 co-expressed gene modules were identified, and each module was marked by a color (Fig. 3A). Each module contained a group of mRNAs with high TOM, which were coordinately expressed and potentially involved in similar biological processes. The grey module contained 3178 genes that were not attributed to any modules. To test the stability of the gene modules, we randomly separated the data set to obtain a train cohort and a validation cohort. All the modules had Z summary scores (Z scores) > 10, meaning they were very conservative (Supplementary Table 1). Figure 4A shows the heatmap plot that represents the entire lncRNA expression network. For the lncRNA co-expression network, 55 samples and 2097 DE-lncRNAs were subjected to WGCNA. Figure 2B shows that when the power was between 16 and 18, the scale-free topology fit index was 0.85. By the dynamic tree cut method, 3 co-expressed gene modules were identified in the aggregate, and each module was marked by a different color (Fig. 3B). Each module contained a group of lncRNAs that were coordinately expressed and had a high TOM, and they potentially involved in similar biological processes. The grey module contained 461 lncRNAs that were not attributed to any modules. We used the same method to test the stability of the lncRNA modules and found that all the modules were highly conservative with Z scores > 10 (Supplementary Table 2). Figure 4B shows the heatmap plot that represents the entire lncRNA expression network.

### Association of the modules with the clinical traits, and identification of the hub protein-coding genes and lncRNAs

**Figure 5:**
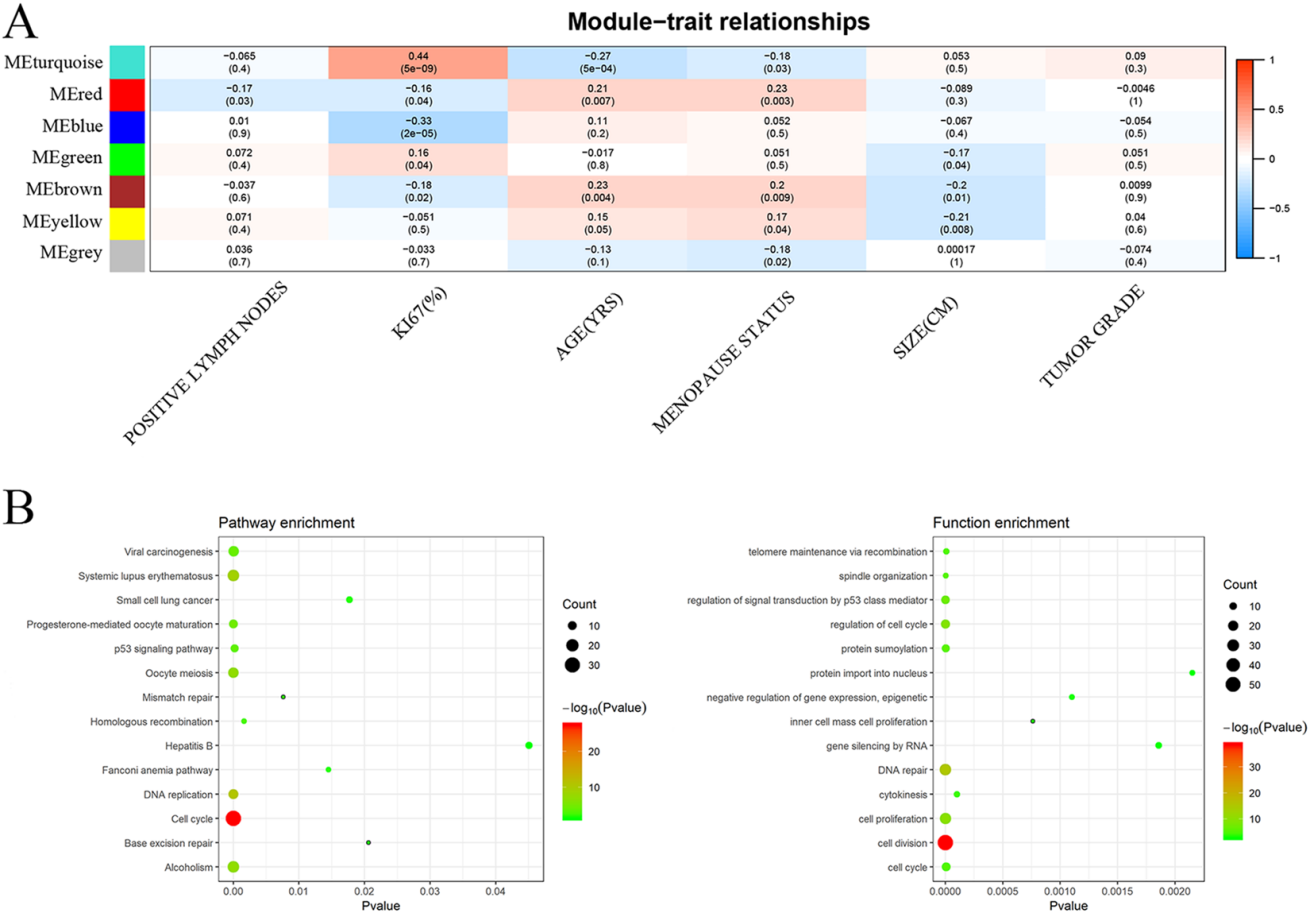
(**A**) Pearson correlation coefficient matrix among the module eigengenes (MEs), and breast cancer characteristics. Each cell reports the correlation (and *P*-value) among the module eigengenes (rows) and traits (columns). (**B**) The KEGG pathway enrichment and GO analyses in the turquoise module. GO = gene ontology, KEGG = Kyoto Encyclopedia of Genes and Genomes.

The correlation between the tumor characteristics and Module eigengenes (MEs) was determined by the interaction analysis of the modules related to a clinical feature (Fig. 5A). The eigengenes of the turquoise module was also highly correlated with Ki-67 status (cor = 0.44, *p* = 5 × 10^−9^). We filtered out the protein-coding genes and lncRNAs that had MM values > 0.8 and MM *P*-value < 0.05 in the modules. These hub genes are exhibited in Supplementary Tables 3 and 4.

### Functional enrichment analysis

The turquoise module was highly correlated with Ki-67 status. Therefore, the GO enrichment and KEGG pathway analyses were carried out to gain an insight into the biological characteristics of this module. For the turquoise module, the genes were mainly concentrated in GO: 0006281 (DNA repair) and GO: 0008283 (cell proliferation). The KEGG pathway analysis of the turquoise module revealed hsa04110 (Cell cycle), hsa05203 (Viral carcinogenesis), and hsa04115 (the p53 signaling pathway). The complete information is shown in Fig. 5B and Supplementary Tables 5 and 6.

### Differentially expressed mRNAs, lncRNAs, and miRNAs in TNBC

**Figure 6:**
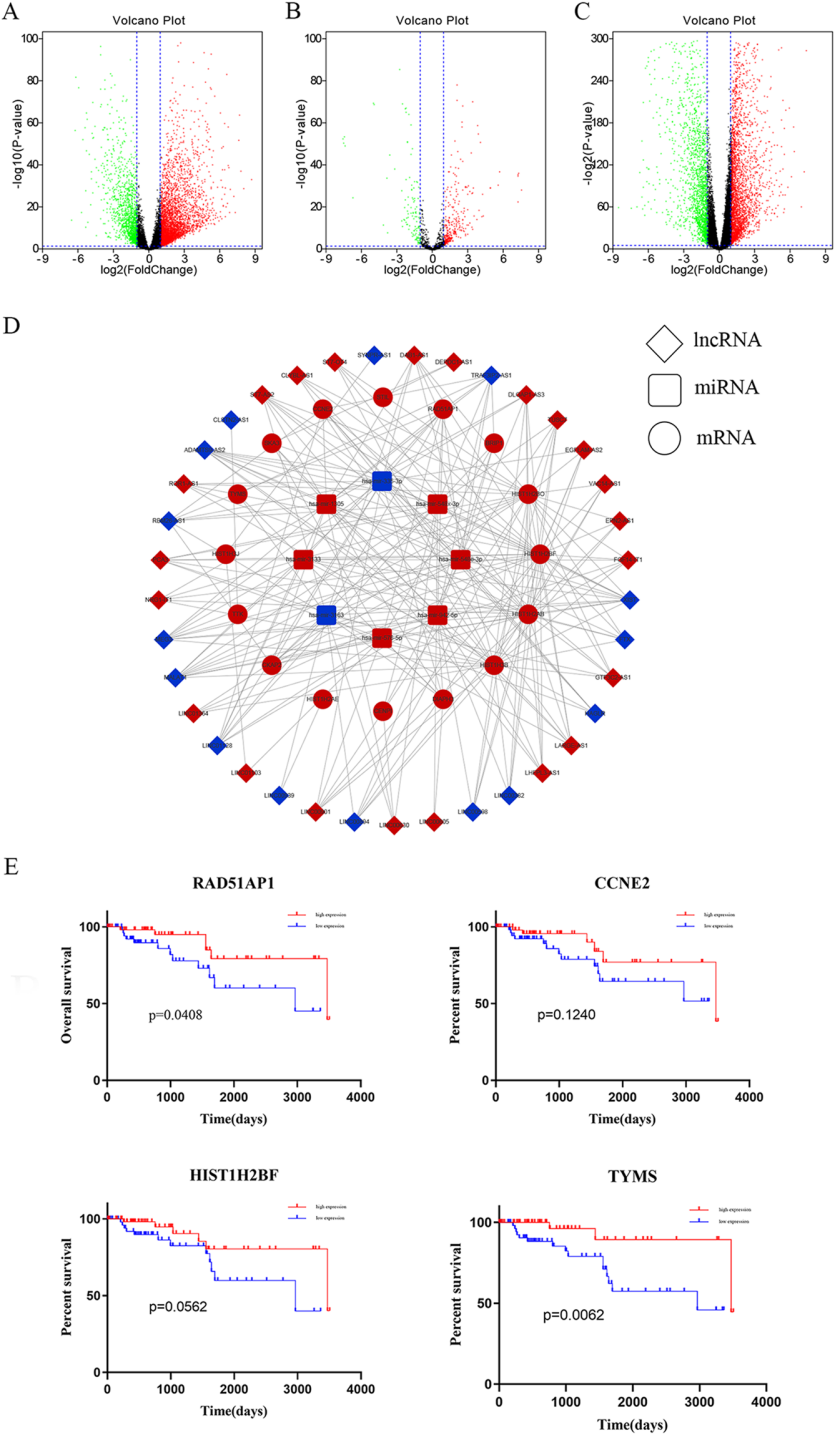
Volcano plots showing the expression profiles of lncRNAs (**A**), miRNAs (**B**), and mRNAs (**C**). (**D**) Global view of the ceRNA network in TNBC. Red and blue depicts up- and down-regulated genes, respectively. (**E**) The Kaplan-Meier curve analysis of the DEmRNA for the overall survival in TNBC.

With the standard thresholds |log2FC| > 1 and *p*-value < 0.05, we identified 4,852 lncRNAs, 427 miRNAs, and 4,565 mRNAs differentially expressed in the TNBC tissues compared with the normal mammary tissues. Some of the differences were larger than 80-fold (Fig. 6A–C).

### Construction of the ceRNA network in TNBC and Kaplan–Meier curve analysis of the mRNAs in the network

LncRNA-miRNA interactions and miRNA-mRNA interactions were combined to establish a complete lncRNA-miRNA-mRNA network, which consisted of 37 lncRNAs, 28 miRNAs, and 16 mRNAs, totaling to 243 interactions (Fig. 6D). The Kaplan-Meier survival analysis was performed to investigate the overall survival according to the mRNA expression pattern. The results demonstrated that high expression of RAD51AP1 and TYMS may be considered a useful prognostic indicator for TNBC patients (Fig. 6E).

## Discussion

The ceRNA concept has been proposed recently. It describes a class of RNAs that has miRNA binding sites and can thus compete with the miRNA-targeted mRNAs for the miRNAs^11^. Understanding of ceRNA crosstalk has shown that miRNAs and their targets establish complex ceRNA networks^21^. Multiple studies have suggested that abnormal lncRNA expression conduces to DNA damage, hyperplasia, and poor prognosis in breast cancer^22–24^. However, a comprehensive analysis of the differential expression profiles of lncRNA and ceRNA networks in TNBC has been lacking.

In this study, we applied the WGCNA to the current microarray mRNA and lncRNA expression data obtained from breast tissues of TNBC patients and provided in the GEO database. We thereby located the hub protein-coding genes and lncRNAs. Among the modules, the turquoise module correlated with Ki-67 status the most. Functional enrichment analysis results showed that the hub genes in the turquoise module were associated with DNA repair, cell proliferation, and the p53 signaling pathway. To gain a better insight into the functions of these hub genes, we performed co-expression analysis of the hub protein-coding genes and lncRNAs in the turquoise module. The RNA-seq data obtained from the TCGA database revealed that the protein-coding genes and lncRNAs that were co-expressed in the TNBC tissues showed a differential expression pattern compared with their expression pattern in the normal mammary tissues. After constructing the ceRNA network, two mRNAs (RAD51AP1 and TYMS) were found to be correlated with overall survival in TNBC. These data indicate that the identified ceRNA network is involved in the formation and development of TNBC.

Recently, miRNAs have extensively been studied. Mechanisms involving miRNAs have been shown to take part in various cancer types, and even the same miRNA can be involved in multiple cancers. For example, in our ceRNA network, hsa-mir-335 and hsa-mir-942 have been shown to play key regulatory roles in a variety of cancers, including epithelial ovarian, lung, and colorectal cancers^25–27^. Furthermore, hsa-mir-335 has been reported as the first selective tumor initiation and metastasis suppressor locus in breast cancer in humans^28^.

Regarding the correlation between the cancer-specific mRNAs and patient prognosis, the results we obtained are not entirely consistent with previous reports. Although high expression of RAD51AP1 has been reported as a biomarker for poor overall survival in lung cancer^29^, there is little known about the function of RAD51AP1 in TNBC. Additionally, CCNE2 and HIST1H2BF were found to have no significant correlation with overall survival in TNBC (p > 0.05). Nevertheless, CCNE2 may play an important mechanistic role in non-small cell lung cancer and breast cancer^30,31^. Unfortunately, there have been few studies about the roles of TYMS and HIST1H2BF in breast cancer. Therefore, further research is needed to clarify the role of these genes in TNBC.

In the cytoplasm, ceRNA-mediated regulatory mechanisms constitute an important pathway for lncRNAs to modulate post-transcriptional regulation. Previous studies have shown that lncRNAs can serve as miRNA “sponges,” and compete with miRNA-targeted mRNAs for miRNAs, thereby affecting the miRNA-mediated gene regulation. The ceRNA network includes the genes that we analyzed by the functional enrichment method, such as TYMS in cell proliferation and RAD51AP1 in DNA repair. In addition, we also found that genes were enriched in multiple TNBC-related pathways, such as CCNE2 in the p53 signaling pathway.

There are several limitations to our study. First, not all the TNBC GEO data were analyzed in this study. Therefore, due to the two predictions, this study unavoidably suffers from selection bias. Moreover, this study lacks biological experimental confirmation. As the next step, we will validate and further investigate the lncRNA-miRNA-mRNA relationships of the ceRNA network alongside biological experiments.

In summary, we have established a ceRNA network in TNBC. Our results suggest that the tumor-specific mRNAs and lncRNAs in TNBC may be involved in a complex ceRNA network, presenting them as potential therapeutic targets for the treatment of TNBC.

## Methods and Materials

### Data collection and pre-processing

The RNA-sequencing (RNA-Seq-HTSeq) and clinical data of The Cancer Genome Atlas (TCGA) BRCA dataset were downloaded from TCGA (https://tcga-data.nci.nih.gov/tcga/) data portal on March 29, 2019. Microarray mRNA and lncRNA expression data were downloaded from the NCBI Gene Expression Omnibus (GEO). The GSE76250 dataset contains mRNA expression data from 33 normal breast and 165 TNBC tissue samples, and the GSE65216 dataset contains lncRNA expression data from 11 normal breast and 55 TNBC tissue samples. The raw CEL files derived from each microarray dataset were normalized by using the R package “limma”. Next, we selected the probes for the mRNAs and lncRNAs from the corresponding microarray platforms. Finally, we obtained 17,644 unique protein-coding genes and 2097 annotated lncRNA transcripts that we used for the following analyses.

### Identification of the differentially expressed protein-coding genes (DEGs) and lncRNAs

Differentially expressed protein-coding genes (DEGs) and lncRNAs (DE-lncRNAs) in TNBC and normal mammary samples were detected by “edgeR” with R package^32^. The thresholds for both were set as *P*-value < 0.05, and the significant residual was used for the WGCNA. Next, the DEGs and DE-lncRNAs with |log_2_ (fold change [FC])| > 1 were identified by Volcano plots by using the R package “ggplot2.”

### Construction and module detection of the weighted gene co-expression network

We used the R package “WGCNA” to construct the protein-coding gene and lncRNA co-expression networks according to the DEGs and DE-lncRNAs, respectively. Firstly, the outlier samples were removed and the hierarchical clustering analysis was performed with the “hclust” R function. Secondly, we used the integral function “pickSoftThreshold” to select the accurate cut-off point. Thirdly, a TOM was transformed from the adjacency. The gene “minModuleSize” was set at 30 to assure high reliability. As for the lncRNA network, since there were few DE-lncRNAs, the minModuleSize was set to 15. Then, to summarize the expression patterns of the module genes into a single characteristic expression profile, each module’s gene expression matrix component, called the module eigengene (ME), was obtained by the WGCNA^33^. The processes followed to construct the lncRNA co-expression networks were similar to the gene co-expression networks.

### Module preservation analysis

Based on the data source of the DEGs or DE-lncRNAs, the R package “caret” was used to generate two datasets named “train” and “test”. Afterward, we used the WGCNA (module preservation) analysis to calculate the Z scores. The Z scores ranging from 2–10 exhibit low-to-moderate preservation and those >10 exhibit high preservation. It should be noted that, regardless of the protein-coding gene or lncRNA modules, the grey module contains protein-coding genes or lncRNAs that are not part of any module, while the gold module is produced through the preservation function during the statistical analysis. Therefore, the preservation analysis results do not show these two modules.

### Modules related to the clinical parameters, and confirmed hub protein-coding genes and lncRNAs

The WGCNA utilizes ME to evaluate the possible connections between the gene modules and clinical traits. In this study, genetic significance (GS) and modular significance (MS) were used to calculate the modular expression patterns associated with the clinical characteristics of the samples. The GS of a gene was defined as the correlation coefficients for different clinical characteristics, and MS was defined as the average GS of all the genes in the module. This analysis was only applied to the protein-coding gene dataset but not to the lncRNA dataset due to the lack of the necessary information about the samples. The hub protein-coding genes were determined by calculating the module membership (MM) and MM *P*-value. The MM of a gene was defined as the correlation between ME and the gene expression profile. We defined genes as hub genes in the modules if they had a value of MM > 0.8 and MM *P*-value < 0.05. The same method was applied to identify the hub lncRNAs.

### Functional annotation of the modules and identification of the hub genes

To explore the functions and pathways related to the hub genes and lncRNAs, we performed the gene ontology (GO) terms and Kyoto Encyclopedia of Genes and Genomes (KEGG) pathways analyses by using the DAVID bioinformatics tool (version 6.8, https://david.nciferf.gov/home.jsp). The threshold was set as *P*-value < 0.05. The DEGs were considered as hub genes if they were extracted from the GO functions or KEGG pathways.

### Construction of the lncRNA-miRNA-mRNA ceRNA network and survival analysis

For validation, RNA and miRNA sequencing data and clinical information were obtained from the TCGA database (https://cancergenome.nih.gov/). The differentially expressed mRNAs, lncRNAs, and miRNAs between the TNBC and normal mammary tissues were analyzed by edgeR package in R language. A gene was defined as differentially expressed (DE) when the *P*-value was <0.05 and the fold change (FC) was ≥2-fold higher or lower (|log FC| > 1). The construction of the ceRNA network included three steps: (1) Co-expression analysis of the hub protein-coding genes (associated with Ki-67%) and lncRNAs in the turquoise module (more lncRNAs); (2) The putative mRNA targets of the miRNAs were predicted by using DIANA-microT-CDs (http://diana.imis.athena-innovation.gr/), and only the miRNAs predicted to bind to four genes were retained; (3) We used DIANA-LncBase v2 (http://diana.imis.athena-innovation.gr/) to predict the putative lncRNA targets of the miRNAs obtained in step (2). The intersection of the predicted lncRNA targets and those obtained in step (1) were used for further analyses. The lncRNA-miRNA-mRNA ceRNA network was established and visualized by using Cytoscape v3.0^34^.

Overall survival time was evaluated via Kaplan-Meier analysis by using GraphPad Prism 7.0 (GraphPad Software, Inc., La Jolla, CA, USA). The log-rank test was used.

## Supplementary information

Supplementary tables

## Data Availability

The RNA-sequencing data and clinical data that support the findings of this study are openly available in The Cancer Genome Atlas at (https://tcga-data.nci.nih.gov/tcga/). The two public gene expression data sets, GSE76250, and GSE65216 can be downloaded from NCBI Gene Expression Omnibus database at (https://www.ncbi.nlm.nih.gov/geo/).
