# Supplementary material for: Gene and lncRNA co-expression network analysis reveals novel ceRNA network for triple-negative breast cancer
Source: Sci Rep. 2019 Oct 22;9:15122. doi: 10.1038/s41598-019-51626-7 (PMC6805880; doi:10.1038/s41598-019-51626-7)
Supplement: Supplementary file 1 — Supplementary tables [file 41598_2019_51626_MOESM1_ESM.pdf]

Gene and lncRNA co-expression network analysis reveals novel ceRNA network for triple-negative breast cancer

Kehao Le[1*], Hui Guo[1*], Qiulei Zhang[1], Xiaojuan Huang[2], Ming Xu[1], Ziwei Huang[1], Pengfei Yi[1,a]

[1] Department of Breast and Thyroid Surgery, Union Hospital, Tongji Medical College, Huazhong University of Science and Technology, Wuhan, China

[2] Department of Nuclear Medicine, Union Hospital, Tongji Medical College, Huazhong University of Science and Technology, Wuhan, China

[*] These authors contribute equally to this work.

[a] Correspondence: Pengfei Yi, Department of Breast and Thyroid Surgery, Union Hospital, Tongji Medical College, Huazhong University of Science and Technology, Wuhan 430022, China.

Email: yipengfei1986@126.com

Supplementary Table 1. Z-score of gene modules

Module	Z-score
blue	47
brown	34
gold	32
green	23
grey	24
red	15
turquoise	40
yellow	48

Supplementary Table 2. Z-score of lncRNA modules

Module	Z-score
blue	1.5
gold	18
grey	13
turquoise	25

Supplementary Table 3. Hub lncRNAs identified in the preserved turquoise and blue.

Modules	Hub lncRNAs
MEturquoise*	RAD51-AS1, LOC283788, EIF1B-AS1, LOC101243545 , ITGA9-AS1, LINC00102, LOC102546226, NCK1-AS1, LOC101928818 , LOC100506844, LHFPL3-AS1, SUGT1P3, WAC-AS1, LOC101928844, LINC00691, LOC101927040, SUGT1P1, LOC101927079 , LOC101928201, LINC00320, LOC100126784, WHAMMP2 , ADD3-AS1, LOC100240728, LOC101927870, LOC286190, LOC100133920 , LINC01085, LOC389834, LOC654342, BRD7P3, ATP8B5P, SH3GL1P1, HOXA-AS2, LOC101929681, LINC00997, SNHG1 , LINC01020, WDR86-AS1, SNHG10, TRAF3IP2-AS1, LINC00485, LINC00266-1, LOC101929239, LINC00844, LOC400620, LOC100133920, LOC101928781, ANKRD20A12P, OR5AK4P
MEblue	BRD7P3, LINC00702

*50 of 459 hub lncRNAs in MEturquoise are listed.

Supplementary Table 4. Hub mRNAs identified in the preserved modules.

Modules	Hub mRNAs
MEgreen	CXCL10, STAT1, CD80, GBP5, CXCL11, LAMP3, EPSTI1, NMI, OAS2, LAP3, IDO1, OASL, RSAD2, ETV7, OAS3, TRIM69, DTX3L, PARP12, RNF213, PARP9, PSMB9, NLRC5, PARP14, OAS1, SAMD9, IFI44, IRF1, IFIH1, GBP7, IFIT3, WARS, HLA-G, HLA-F, IFI44L, IFIT2, DDX60, SAMD9L, HLA-C, GBP4, HLA-B, HLA-A, CD274, IFI35, GBP1, UBE2L6
MEbrown	FPR3, CD86, LAPTM5, TFEC, CTSS, HAVCR2, PIK3AP1, IGSF6, CTSC, CD84, NPL, NCF2, TLR8, CD68, MNDA, LCP1, PLEK, THEMIS2, EVI2A, CCR1, TYROBP, CYBB, ARRB2, CD4, GPR65, CECR1, BIN2, FYB, LPXN, NCKAP1L, CD53, VAV1, LY86, RHOG, PTAFR, BTK, HCK, CXorf21, CLEC4A, LCP2,

	SCIMP, MYO1F, C3AR1, RNASE6, LAIR1, EVI2B, C1QB, SRGN, HCST, C1QA, LGMN, IL10RA, DOCK2, DPYD, LY96, C1orf162, CD163, ALOX5, ADAP2, HLA-DRB3, AOAH, HLA-DMB, NCF4, SELPLG, HLA-DMA, SLC8A1, CYTH4, LAIR2, SLCO2B1, HLA-DRB1, HCLS1, LRRC25, TPP1, MILR1, TBXAS1, MPEG1, CD14, CASS4, GMFG, SH2B3, AIF1, SAMHD1
**MEred**	BGN, CTHRC1, FN1, COL1A1, LRRC15, COL3A1, COL5A2, MMP14, COL5A1, COL1A2, BMP1, ADAMTS2, SPARC, MXRA5, CDH11
**MEblue**	ABCA10, CTTNBP2, FXYD1, LDB2, ABCA9, TTC28, PGM5, NOVA1, ARHGAP6, KIAA1377, KL, SDPR, ACOX2, TENC1, FAM162B, FAM13C, ARHGAP20, TSHZ2, GSN, FAXDC2, JAM2, ZNF385D, MOCS1, FIGF, RHOJ, LEPR, ALDH1A2, LRRN4CL, CEP112, FGF10, TSPAN7, PPAP2B, PDGFD, FAT4, EBF1, CD34, PLSCR4, ABCA6, MEOX2, GPR124, GPC3, GIPC2, ABCA8, FHL1, ACACB, CCL14, AGTR1, EBF3, SHE, BMX, MMRN2, DLC1, EMCN, LAMA2, EBF2, RAMP2, ZNF423, CASQ2, ASPA, TNS1, CXorf36, TMEM88, NPR1, ERG, CXCL12, DCLK1, SH3D19, KANK2, ITIH5, RECK, MYCT1, LPAR1, MFAP4, ANGPTL1, LHFP, ECSCR, TBX15, FHL5, GNG11, FLRT2, CYBRD1, SLIT3, ADH1C, SPARCL1, CFD, FAM198B, TFPI, S1PR1, PPARG, CDO1, ANK2, CPED1, AKAP12, INMT, ADH1A, TEK, ADH1B, OLFML1, FILIP1L, SRPX, TNXB, EDNRB, CLEC14A, GAS7, DPT, ZCCHC24, OGN, CDH5, IL33, LRP1, DCN, CHRDL1, FMOD, CNRIP1, VWF, ANTXR2, EHD2, PODN, ABI3BP, CDK15, TWIST2, FABP4, RFTN2, COL14A1, AOX1, MAP1A, GPX3, MAF, STARD8, PCSK5, IGFBP4, HSPB6, PPAP2A, ZEB1, IGF1, ITSN1, LYVE1, ZEB2, CRTAP, CRHBP, GNG2, CST3
**MEyellow**	ICOS, CCR4, FERMT3, SNX20, CTLA4, ARHGAP30, CD72, MYO1G, SELL, CD37, IRF8, CCR7, SASH3, CD52, TRAF3IP3, C5orf20, FMNL1, CD48, IL21R, P2RY8, LCK, JAK3, CD6, SLA2, GZMK, WDFY4, STK10, DENND1C, CCR5, GPR171, WAS, EOMES, PARVG, CCL5, RASSF5, STAP1, CD5, GPR18, CD226, LTB, CAMK4, CST7, ITGB7, CD247, TRAT1, SLAMF6, NKG7, SIRPG, CYTIP, SIPA1, FCRL3, RAC2, TAGAP, IL2RB, CARD11, ARHGAP9, TRAV1-2, ACAP1, ICAM3, GZMH, SH2D1A, ZBP1, PRKCB, TIGIT, HLA-DQB1, CD2, RASAL3, CD38, SP140, PLAC8, FAM78A, NCF1, FAIM3, HMHA1, KLHL6, MAP4K1, IKZF3, TRAF1, CD3G, ITK, SAMD3, PARP15, CD8A, UBASH3A, CD96, PRF1, IL2RG, KLRG1, IKZF1, PLCB2, GRAP2, CD3E, ZNF831, ZAP70, TMC8, BANK1, PYHIN1, CLEC2D, BTLA, ARHGAP15, LAX1, CD69, CD27, ARHGAP25, CRTAM, ANKRD44, CCR2, IPCEF1, CCR6, C16orf54, DOCK10, FGD2, WIPF1, CXCR6, LY9, GIMAP2
**MEturquoise**	NUSAP1, MKI67, TOP2A, ANLN, CKAP2, HIST1H3B, KIF11, CCNA2, CEP55, LMNB1, ARHGAP11A, ARHGAP11B, NCAPG, KPNA2, BUB1, CENPE, PRR11, PLK1, CENPF, CDK1, CKAP2L, CRY2, HIST1H2AM, CASC5, CENPN, KIF20A, NDC80, FAM72D, DTL, SKA3, H2AFZ, AURKA, HIST1H2BO, HIST1H2BF, KIF4A, KIF2C, KIF23, TTK, PRC1, CLSPN, MELK, CKS2, CCNB2, SMC4, STIL, RACGAP1, EZH2, HIST1H3D, CENPA, KIF14, HJURP, CCNB1, PAK6, HIST1H2AH, UBE2T, NCAPH, STMN1, BLM, HIST1H2BJ, ECT2, HIST1H2AE, EXO1, FAM72B, DBF4, CENPO, CDC6, SGOL1, UBE2C, E2F3, FAM72A,

		MYBL2, MAD2L1, FEN1, MCM10, BIRC5, ATAD2, SRPK1, HDGF, PBK, FAM83D, HIST1H2AB, KIF15, KIF18A, CDC7, GINS1, CDC25C, RAD51, MCM4, PCNA, MCM6, KIAA1524, FOXM1, CDC123, DIAPH3, POLQ, CENPL, WDHD1, BRIP1, DEPDC1, CDC20, IQGAP3, TRIP13, CENPI, SMG5, SGK494, PTTG1, CDC45, NUDT5, SKA1, RAD51AP1, TYMS, UQCRHL, CHEK1, ORC1, NEK2, RAD54L, TICRR, CDCA8, RNASEH2A, METTL13, OIP5, MASTL, CDCA2, DNA2, PLK4, HIST1H2BN, DONSON, SGOL2, MND1, CDC25A, ILF2, DEPDC1B, XRCC2, AURKB, SMC2, ZNF695, CTPS1, ORC6, INCENP, CHAF1B, MCM2, CCNE2, PRIM2, KIF22, RQCD1, RFWD3, NUDCD1, CDCA3, NCAPG2, PRCC, ATAD5, CKAP5, PTMA, C1orf131, XPO5, NDC1, TOPBP1, FBXO5, GMPS, MCM7, SUV39H2, PFDN2, LIN9, DSCC1, SNRPD1, C1orf112, URB2, RAD21, SNRPG, UCK2, RAD54B, SNRPC, GPSM2, TBCE, GTPBP4, MCM3, RFC4, SNRPA1, SASS6, TCF19, DHX9, CKS1B, DKC1, BYSL, CDK2, USP1, ANP32E, BARD1, SSRP1, SEPHS1, HIST1H3J

Supplementary Table 5. Gene Ontology enrichment analysis of genes in turquoise module.

Term	Pathway	Pvalue	Count
GO:0051301	cell division	2.91E-39	56
GO:0008283	cell proliferation	1.47E-10	27
GO:0051726	regulation of cell cycle	1.03E-09	16
GO:1901796	regulation of signal transduction by p53 class mediator	7.76E-08	14
GO:0016925	protein sumoylation	2.35E-06	12
GO:0007051	spindle organization	3.51E-06	6
GO:0007049	cell cycle	8.90E-06	15
GO:0000722	telomere maintenance via recombination	9.17E-06	7
GO:0000910	cytokinesis	1.01E-04	7
GO:0001833	inner cell mass cell proliferation	7.61E-04	4
GO:0045814	negative regulation of gene expression, epigenetic	0.001099737	6
GO:0031047	gene silencing by RNA	0.001856988	8
GO:0006606	protein import into nucleus	0.002148204	6
GO:0006281	DNA repair	6.48E-16	28

Supplementary Table 6. The KEGG pathways of genes in turquoise module.

Term	Pathway	Pvalue	Count
hsa04110	Cell cycle	5.07E-28	32
hsa03030	DNA replication	1.57E-11	12
hsa05322	Systemic lupus erythematosus	1.41E-09	17
hsa04114	Oocyte meiosis	6.90E-08	14
hsa05034	Alcoholism	8.06E-08	17
hsa04914	Progesterone-mediated oocyte maturation	2.21E-05	10
hsa05203	Viral carcinogenesis	6.84E-05	14
hsa04115	p53 signaling pathway	1.76E-04	8
hsa03440	Homologous recombination	0.001635591	5
hsa03430	Mismatch repair	0.007608572	4

hsa03460	Fanconi anemia pathway	0.014519525	5
hsa05222	Small cell lung cancer	0.017727563	6
hsa03410	Base excision repair	0.020600369	4
hsa05161	Hepatitis B	0.045072484	7
